# Molecular Homeostasis Failure and Pathological Integration: Bridging Molecular Perturbations with Disease Phenotypes

**DOI:** 10.3390/ijms27062593

**Published:** 2026-03-12

**Authors:** Kazuhiko Nakadate

**Affiliations:** Department of Functional Morphology, Meiji Pharmaceutical University, 2-522-1 Noshio, Kiyose 204-8588, Tokyo, Japan; nakadate@my-pharm.ac.jp

## 1. Introduction

The molecular basis of human disease is increasingly recognized as a consequence of disrupted homeostatic regulation rather than isolated genetic mutations or single biochemical abnormalities [1]. Living systems are inherently dynamic and resilient, supported by multilayered regulatory networks that coordinate redox balance, protein quality control, metal ion homeostasis, energy metabolism, and cellular clearance [2]. These networks enable tissues to tolerate substantial molecular perturbations without manifesting overt pathology [3]. Disease phenotypes typically arise not from the mere presence of molecular abnormalities, but from the gradual erosion of compensatory capacity and the eventual collapse of system-level resilience [1]. Importantly, this collapse is often silent until a critical threshold is exceeded. Pathology therefore represents a cumulative and integrative record of molecular stress over time, capturing both adaptive responses and irreversible damage [2].

This Special Issue of the *International Journal of Molecular Sciences* aims to emphasize the importance of integrating molecular discoveries with experimental pathology to better understand how disturbances in homeostatic regulation are translated into tissue- and organ-level disease phenotypes. Such integration is essential for advancing mechanistic insight and translational relevance in contemporary molecular medicine.

## 2. Overview of the Special Issue Contributions

Recent advances in molecular biology, genomics, and metabolomics have dramatically expanded our ability to detect disease-associated molecular alterations [4]. However, a fundamental challenge remains: molecular changes do not necessarily equate to pathological significance [5]. In many experimental and clinical contexts, substantial molecular perturbations coexist with preserved tissue architecture and function, reflecting the presence of robust compensatory mechanisms [6].

Cells respond to molecular stress through coordinated adaptive programs, including activation of antioxidant defenses, enhancement of protein degradation pathways, metabolic rewiring, and modulation of inflammatory signaling [7]. These responses define a compensatory phase during which pathology remains minimal or absent. The transition to overt disease often occurs abruptly, when multiple stressors converge and overwhelm adaptive capacity [8]. Pathological phenotypes emerge from complex, non-linear interactions among oxidative stress, protein misfolding, mitochondrial dysfunction, and metal dyshomeostasis [9]. Experimental pathology provides a unique framework for identifying the points at which these interacting perturbations become biologically consequential, revealing thresholds of failure that are invisible at the molecular level alone [9].

Experimental pathology serves as an indispensable interpretive framework for contextualizing molecular findings. Histopathological evaluation reveals tissue-specific vulnerability, regional heterogeneity, and characteristic patterns of cellular injury that are not captured by bulk molecular analyses [10]. Immunohistochemistry further allows precise localization of molecular alterations to defined cell populations, while ultrastructural analyses uncover subcellular damage affecting organelles such as mitochondria, lysosomes, and the endoplasmic reticulum [11,12,13].

Particularly informative are organs involved in systemic surveillance and clearance, including the liver, spleen, and reticuloendothelial system [14,15]. These organs integrate signals from circulation, metabolism, and immune responses, making them sensitive indicators of systemic molecular imbalance [16]. Alterations in macrophage activation, erythrocyte turnover, and deposition of oxidative or metal-associated byproducts often precede overt functional impairment [17]. By anchoring molecular changes to spatially resolved tissue phenotypes, experimental pathology enables a more accurate assessment of disease mechanisms and therapeutic impact. Redox regulation represents a central axis linking molecular stress to pathological damage. Reactive oxygen species play essential physiological roles in signaling and host defense; however, sustained redox imbalance disrupts cellular integrity, damages macromolecules, and alters inflammatory and stress-response pathways [18,19]. Metal ions, particularly iron and other redox-active elements, critically modulate these processes by catalyzing oxidative reactions and influencing enzymatic and mitochondrial function [9].

From a pathological perspective, dysregulated redox and metal homeostasis often manifest as reproducible tissue patterns, including pigment deposition, inflammatory infiltration, and cellular degeneration [20]. In my own review contribution to this Special Issue, these mechanisms were examined in detail, emphasizing how oxidative and metal-associated molecular disturbances propagate across cellular networks to produce characteristic pathological phenotypes. Such observations reinforce the concept that oxidative stress and metal dysregulation are not merely secondary consequences of disease but can act as primary drivers of pathological progression when homeostatic control fails [21]. For example, recent work has demonstrated that selective outer mitochondrial membrane (OMM) autophagy mediated by FUNDC1 plays a critical role in maintaining mitochondrial integrity and regulating inflammatory responses, highlighting how organelle-level quality control contributes to tissue resilience [21]. Recent experimental studies have further demonstrated that impairment of mitochondrial quality control mechanisms, including dysregulated mitophagy and stress signaling, directly contributes to inflammatory activation and tissue dysfunction, providing concrete examples of how organelle-level network failure progresses to disease [21].

Natural compounds provide particularly valuable tools for probing the boundaries between physiological adaptation and pathological failure [22]. Tea-derived polyphenols, including catechins and theaflavins, have been extensively studied for their antioxidant, anti-inflammatory, and cytoprotective properties [23,24]. At the molecular level, these compounds modulate redox homeostasis, chelate transition metals, and regulate stress-responsive signaling pathways. Crucially, pathological analyses have demonstrated that such molecular activities translate into tissue-level effects, including preservation of cellular architecture, attenuation of inflammatory responses, and modulation of stress-sensitive cell populations [25]. These findings underscore the importance of evaluating bioactive compounds through integrated molecular and pathological approaches. From this perspective, natural compounds function not only as therapeutic candidates but also as mechanistic probes that illuminate how modulation of molecular networks influences tissue resilience and vulnerability [26]. A recent comprehensive review further detailed the anti-inflammatory mechanisms of plant-derived compounds across cardiovascular, metabolic, neurodegenerative, oncological, and dermatological diseases, emphasizing their modulation of NF-κB signaling, oxidative stress, and inflammasome pathways.

An emerging paradigm in disease biology is the recognition that pathological progression reflects cumulative network failure rather than dysfunction of isolated molecular targets [27]. Biological systems rely on redundancy and cross-regulation to maintain stability; however, aging, environmental stressors, and chronic insults progressively erode this redundancy [27,28]. Pathological phenotypes can thus be understood as emergent properties of compromised network integrity [29]. Integrating systems biology and network pharmacology with experimental pathology provides a powerful framework for identifying critical nodes of vulnerability. Pathological endpoints serve as essential benchmarks for evaluating whether molecular interventions restore functional resilience or merely suppress individual pathways without meaningful tissue-level benefit [30].

The contributions collected in this Special Issue encompass a diverse array of experimental and analytical approaches, including mechanistic in vitro studies, in vivo disease models, quantitative pathology, and integrative computational analyses. A defining feature is the emphasis on mechanistic clarity supported by rigorous pathological validation. Studies that demonstrate coherence among molecular perturbations, cellular responses, and tissue-level outcomes are particularly emphasized. By prioritizing such integrative work, this Special Issue seeks to advance molecular science toward a deeper and more context-aware understanding of disease mechanisms.

## 3. Concluding Remarks

Molecular pathology occupies a pivotal position at the interface of molecular science and clinical medicine. By integrating molecular perturbations with pathological phenotypes, it provides insight not only into how diseases arise, but also into why specific tissues and organs exhibit selective vulnerability. Through this Special Issue, we aim to foster interdisciplinary dialogue and encourage research strategies that align molecular precision with pathological relevance. Such integrative approaches are essential for translating molecular discoveries into meaningful advances in disease understanding, prevention, and therapy.
